# Hsa-miR-181a-2-3p inhibits the oncogenicity of colon cancer by directly targeting STING

**DOI:** 10.18632/aging.206059

**Published:** 2024-08-09

**Authors:** Bowei Liu, Kai Lu, Lijie Yuan, Xiaofang Li, Ling Lan, Shuangyin Han

**Affiliations:** 1 Department of Gastroenterology, Zhengzhou University People’s Hospital, Henan Provincial People’s Hospital, Zhengzhou, Henan 450003, China; 2 Clinical Medicine College, Xinxiang Medical University, Xinxiang, Henan 453000, China

**Keywords:** miR-181a-2-3p, STING, colon cancer, proliferation

## Abstract

Objective: Colon cancer is a common malignant tumor of the gastrointestinal system, which is characterized by high morbidity and mortality. The purpose of this study was to analyze the expression and biological role of miR-181a-2-3p in colon cancer and to investigate the molecular mechanism of its regulatory effect on colon cancer through stimulator of interferon genes (STING).

Methods: Real-time reverse transcription polymerase chain reaction (qRT-PCR) assay was used to detect the expression of miR-181a-2-3p in colon cancer cell lines and normal intestinal epithelial cells. After overexpression of miR-181a-2-3p in colon cancer cell lines SW480 and HT29, cells were examined by CCK8, Transwell, and flow cytometry assays for alterations in proliferation, migration, apoptosis, and cell cycle. Target genes of miR-181a-2-3p were predicted by bioinformatics and validated by dual luciferase assays. Rescue experiments were performed to explore the role of STING in the effect of miR-181a-2-3p. The effect of miR-181a-2-3p on colon cancer proliferation *in* *vivo* was validated by nude mouse tumorigenicity assay.

Results: miR-181a-2-3p was lowly expressed in both colon cancer tissues and cell lines. Overexpression of miR-181a-2-3p led to reduced proliferation and migration, increased apoptosis, and altered cell cycle in colon cancer cell lines SW480 and HT29. STING was a target gene of miR-181a-2-3p. Increased STING expression partially counteracted the effect of overexpression of miR-181a-2-3p on colon cancer cell lines. miR-181a-2-3p also suppressed colon cancer proliferation *in* *vivo*.

Conclusion: miR-181a-2-3p inhibits the proliferation and oncogenicity of colon cancer, and its molecular mechanism could be inhibited by STING.

## INTRODUCTION

Colon cancer is one of the most prevalent common cancers affecting humans worldwide. Unhealthy lifestyle, such as high-fat and low-fiber diet, lack of exercise, smoking and obesity, dysbiosis of the gut microbiota, and family genetics are etiological factors that contribute to the development of colon cancer [1]. According to the 2020 Global Cancer Statistics 2020 Statistical Assessment of Cancer [2] assessment, colorectal cancer ranks third in terms of incidence and second in terms of mortality in the distribution of cancer cases and deaths., in the distribution of cancer cases and deaths, colorectal cancer ranks third in incidence and second in mortality. It is estimated that 106,180 people will be diagnosed with colon cancer and 52,580 people will die from colon cancer in the United States in 2022 [3]. Compared to developed countries, the incidence of colon cancer in China is low, but there is a clear trend of increasing incidence and growing economic burden but its incidence is showing a significant upward trend, and the economic burden is increasing [4].

Colon cancer is characterized by low early diagnosis rate and insignificant symptoms. Early diagnosis depends on screening and patients’ active compliance to screening. Some patients exhibit poor treatment outcomes and high rates of recurrence and metastasis. The presence of micro-metastatic disease micrometastases at the time of surgery is responsible for the majority of main cause of deaths in patients with local and regional colon cancer [5]. Adjuvant chemotherapy and radiotherapy are required for patients at risk of recurrence; patients at risk of recurrence need adjuvant chemotherapy and radiotherapy, however, they can cause considerable side effects, such as peripheral neuropathy due to chemotherapeutic drug toxicity and radiation-induced skin inflammation due to radiotherapy. Therefore, we need to identify better biomarkers and therapeutic targets to diagnose and treat colon cancer.

MicroRNAs (miRNAs) are small RNA polymers of 18 to 24 nucleotides in length that regulate the translation and stability of specific target mRNAs [6]. miRNAs exist in and around cancer-related genomic hotspots of the cancer-related genome, and can function as oncogenes or tumor suppressor genes in the process of carcinogenesis [7]. The potential of miRNAs in cancer diagnosis, prognosis, and treatment is currently receiving increasing attention. Studies have shown the potential value of miR-181a-2-3p in the treatment and prognosis of some cancers. For example, miR-181a-2-3p/let-7i-5p counteracted the EGF pathway by inhibiting SOX2; thereby reducing the number of cancer stem cells in cervical cancer [8]. miR-181a-2-3p negatively regulates the stem cell-like properties of CD44+ ovarian cancer stem cells by targeting EGR1 [9]. miR-181a-2-3p is an oncogenic miRNA in pancreatic cancer and is located at the SNP site rs3802266 of the oncogene ZHX2 3′UTR creating a stronger binding site for miR-181a-2-3p, which has a stronger binding site with miR-181a-2-3p, thus increasing the risk of pancreatic cancer in the Chinese population [10]. miR-181a-2-3p inhibits the growth of gastric cancer and suppresses resistance to cisplatin [11]. Analysis of data from the Cancer Genome Atlas (TCGA) database revealed that two miRNA signatures (hsa-miR-181a-2-3p and hsa-miR-138-1-3p) may be potential indicators of the prognosis of papillary thyroid cancer (PTC) [12]. miR-181a-2-3p predicts recurrence-free survival of patients with follicular variant of papillary thyroid carcinoma (FVPTC) [13]. Serum levels of miR-181a-2-3p can predict the response to preoperative radiotherapy for locally advanced esophageal squamous cell carcinoma (ESCC) and help to personalize the treatment [14].

In the present study, we identified miR-181a-2-3p as an anti-oncogene involved in colon cancer. In addition, miR-181a-2-3p was lowly expressed in colon cancer. Overexpression of miR-181a-2-3p led to reduced proliferation and migration, increased apoptosis, and altered cell cycle in colon cancer cell lines SW480 and HT29. Our study revealed a novel mechanism involved in miR-181a-2-3p/STING-regulated colon cancer.

## RESULTS

### miR-181a-2-3p is lowly expressed in colon cancer

**Figure 1:**
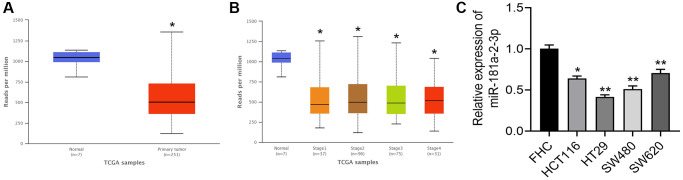
**miR-181a-2-3p is lowly expressed in colon cancer.** (**A**) Expression of miR-181a-2-3p in colon cancer compared with normal. (**B**) Expression levels of miR-181a-2-3p in different stages of colon cancer tissues. (**C**) The relative expression level of miR-181a-2-3p in different cells was detected by q-PCR. The expression level of normal colon cells (FHC) was set as 1. Note: Figure A and B are based on analysis of TCGA database (http://ualcan.path.uab.edu/index.html). Figure 1A: red is the colon cancer group, and blue is the normal control group. Figure 1B: blue is the normal control group, and the rest are colon cancer tissues at different stages.

We analyzed miR-181a-2-3p expression in colon cancer tissues based on the TCGA database, and the results showed that miR-181a-2-3p was lowly expressed in colon cancer tissues (Figure 1A) and miR-181a-2-3p showed low expression in colon cancer tissues at different stages (Figure 1B). Further, we detected miR-181a-2-3p expression in colon cancer cells by qRT-PCR assay, and the results showed that miR-181a-2-3p expression was differentially low in colon cancer cell lines (HCT116, HT29, SW480, and SW620) relative to colon epithelial cells FHC (Figure 1C).

### miR-181a-2-3p affects the biological function of colon cancer cells and miR-181a-2-3p inhibits the oncogenicity of colon cancer

**Figure 2:**
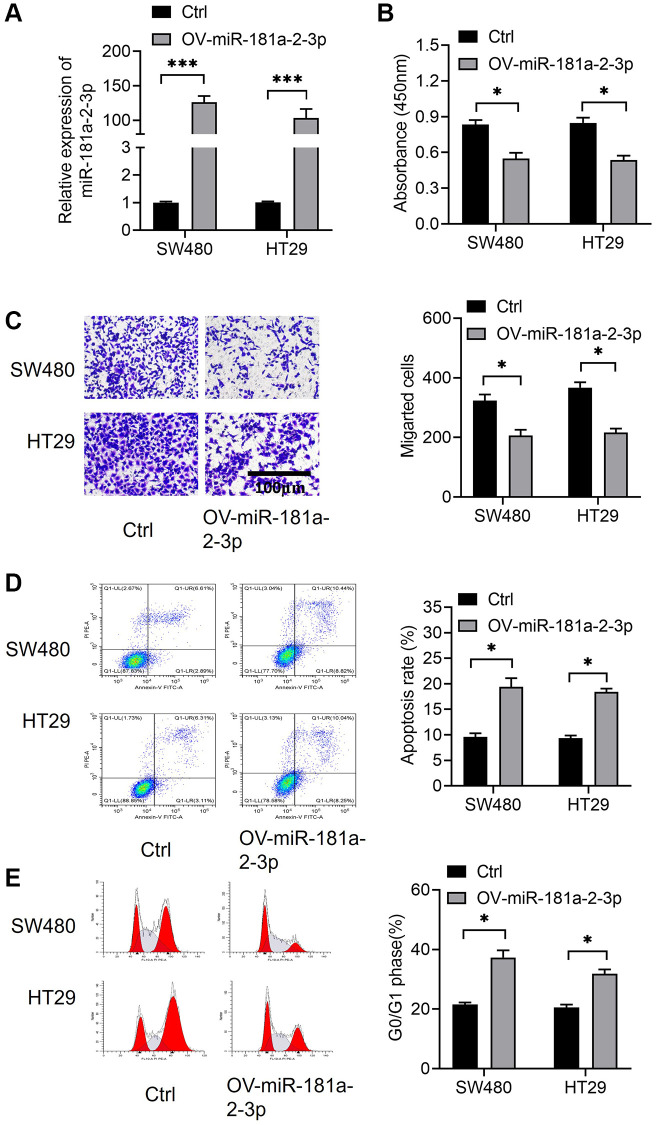
**miR-181a-2-3p affects the biological function of colon cancer cells and miR-181a-2-3p inhibits the oncogenicity of colon cancer.** (**A**) Assessment of miR-181a-2-3p overexpression efficiency by the qRT-PCR method. (**B**) Examination of cell proliferation by CCK-8 assay. (**C**) Assessment of cell migration ability by Transwell assay. (**D**) Measurement of cell apoptosis by flow cytometry. (**E**) Detection of cell cycle by flow cytometry.

We constructed a model of miR-181a-2-3p overexpression by transfecting miRNA mimics in colon cancer cells SW480 and HT29, and verified the efficiency of miR-181a-2-3p overexpression by qRT-PCR assay (Figure 2A). Next, overexpression of miR-181a-2-3p resulted in a decrease in the proliferative capacity of cells on using the CCK-8 assayCCK-8 assay showed that overexpression of miR-181a-2-3p reduced cell proliferation (Figure 2B). Subsequently, the results of Transwell assay revealed that the number of invasive cells in the miR-181a-2-3p mimics group was significantly lower than that in the normal control (NC) group using the Transwell assay (Figure 2C), indicating that miR-181a-2-3p inhibited the migrative ability of colon cancer cells. We also investigated cell apoptosis and cell cycle by flow cytometry. The results revealed that cell apoptosis (Figure 2D) and G1 phase (Figure 2E) were increased in both SW480 and HT29 cells overexpressing miR-181a-2-3p. However, in the G2 phase (Figure 2E), overexpression of miR-181a-2-3p inhibited cell apoptosis. This may be related to the time process of small RNA overexpression, and we focus on the G1 phase.

### miR-181a-2-3p inhibits colon cancer *in vivo* and targets stimulator of interferon genes (STING)

**Figure 3:**
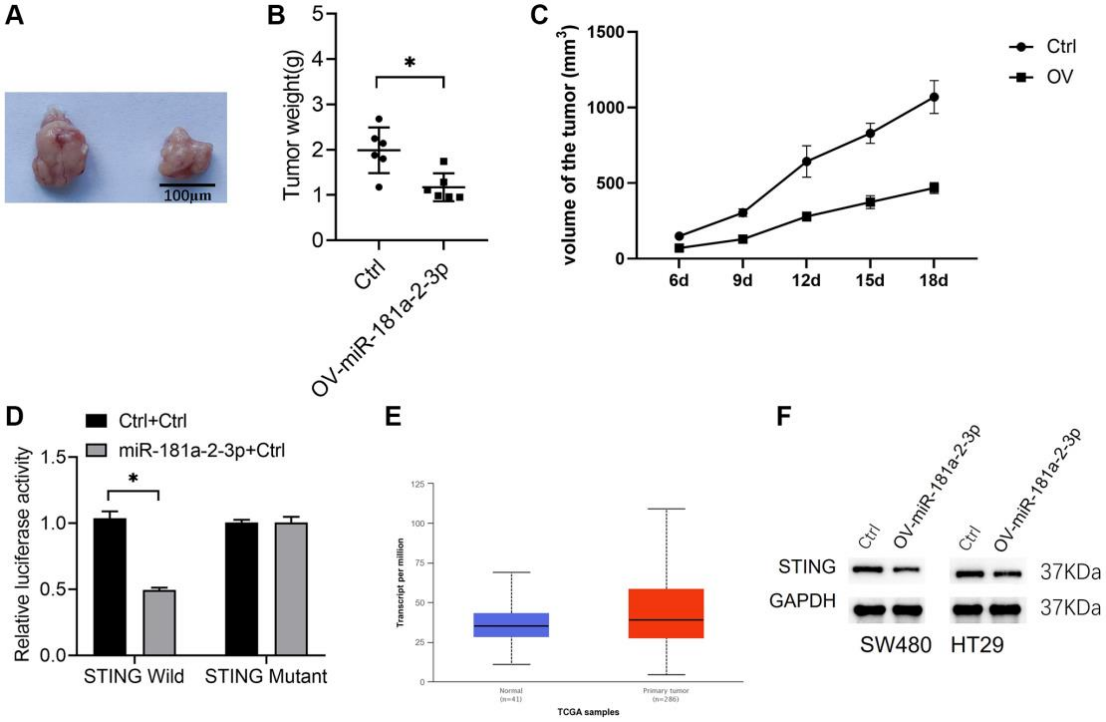
**miR-181a-2-3p inhibits colon cancer *in vivo* and targets *STING.*** (**A**) A nude mouse tumorigenic assay was used to assess the *in vivo* tumorigenic ability of colon cancer. (**B**) Weight of isolated tumors. (**C**) The volume change of the tumor. (**D**) A dual luciferase assay to measure miR-181a-2-3p and STING 3′-UTR regions. Note: The Y-axis is relative luciferase activity or expression, the ratio of Firefly Luciferase (luc2) and Renilla Luciferase (hRluc) to values in Wells of the same sample represents the relative luciferase expression. (**E**) STING expression was analyzed in colon cancer tissues based on the TCGA database. (**F**) Western blot analysis of STING expression in colon cancer cell lines overexpressing miR-181a-2-3p. Note: OV-miR-181a-2-3p is miR-181a-2-3p overexpression group. Ctrl is the control.

We investigated the effect of miR-181a-2-3p on colon cancer growth *in vivo* by the tumorigenesis assay in nude mice. The results showed that isolated tumors from the miR-181a-2-3p overexpression group were smaller than those from the Ctrl group (Figure 3A–3C). Also, we found that *STING* might be a target gene of miR-181a-2-3p (http://mirdb.org/cgi-bin/target_detail.cgi? Target ID = 2267397). Then, we verified the binding effect of miR-181a-2-3p and *STING* 3′-UTR regions by a dual luciferase assay (Figure 3C, 3D). Meanwhile, we analyzed *STING* expression in colon cancer tissues based on the TCGA database, and the results showed that *STING* was overexpressed in colon cancer tissues (Figure 3D, 3E). Moreover, we also performed Western blot analysis examined the expression level of STING expression in colon cancer cell lines overexpressing miR-181a-2-3p by Western blot assay and found that STING protein expression the expression of STING protein was decreased in SW480 and HT29 cell lines overexpressing miR-181a-2-3p (Figure 3E, 3F).

### STING reverses the biological role of miR-181a-2-3p in colon cancer cells

**Figure 4:**
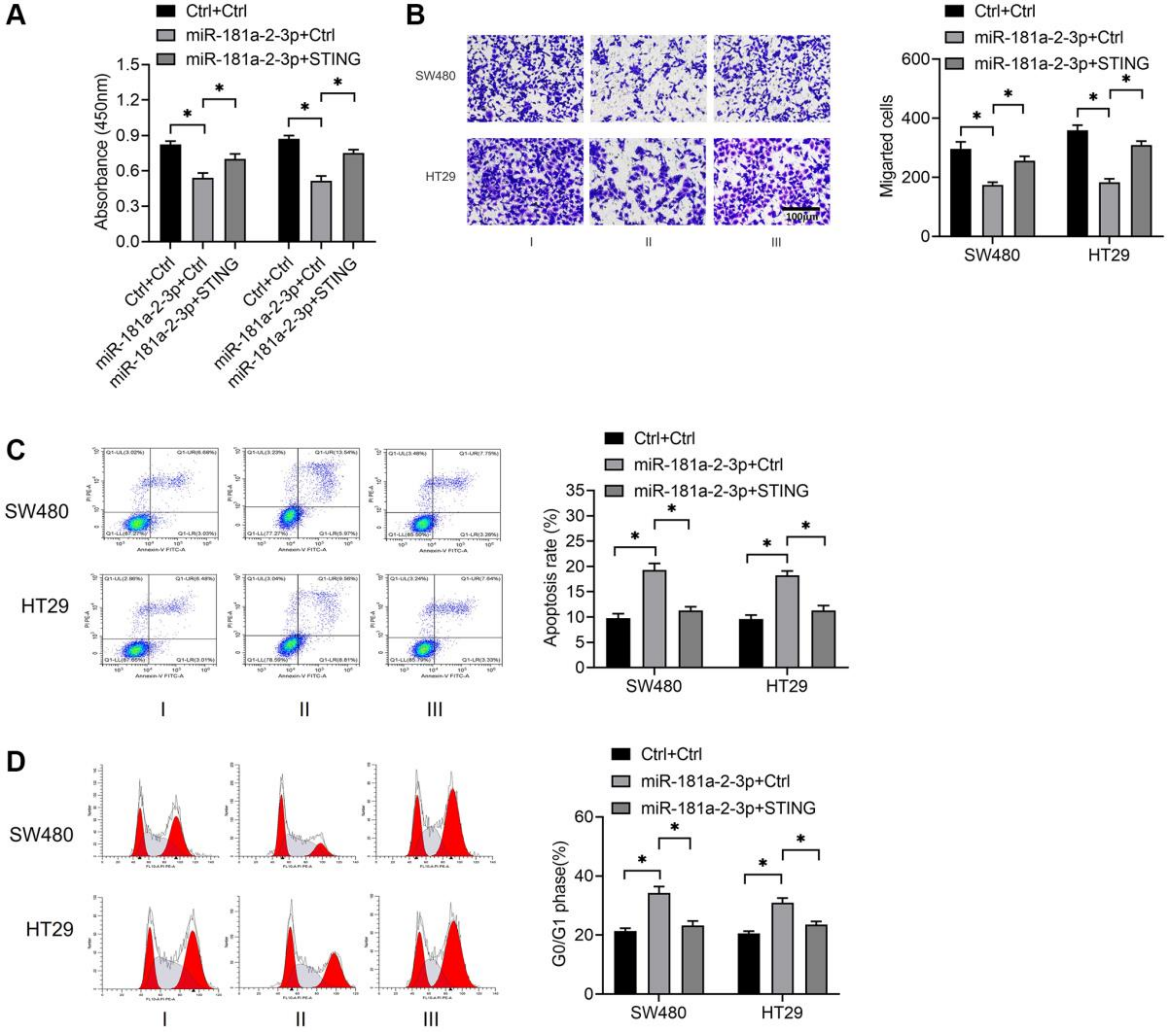
**STING reverses the biological role of miR-181a-2-3p in colon cancer cells.** (**A**) Examination of cell proliferation by CCK-8 assay. (**B**) Assessment of cell migration ability by Transwell assay. (**C**) Measurement of cell apoptosis by flow cytometry. (**D**) Detection of cell cycle by flow cytometry. Note: Group I was Ctrl+Ctrl, which meant that NC overexpressing miR-181a-2-3p and NC overexpressing STING were co-transfected at the same time. Group II was miR-181a-2-3p+ Ctrl, which means that overexpression of miR-181a-2-3p and NC overexpressing STING were simultaneously co-transfected. Group III was miR-181a-2-3p+STING, which means that overexpression of miR-181a-2-3p and overexpression of STING plasmid were simultaneously transfected.

To investigate the role of STING in colon cancer, we simultaneously overexpressed miR-181a-2-3p along with and STING and then detected cell proliferation, migration, apoptosis, and cycle events. The results revealed that STING overexpression significantly reversed the biological effects of miR-181a-2-3p overexpression, including the inhibition of proliferation (Figure 4A) and migration (Figure 4B), the promotion of apoptosis (Figure 4C), and change in the alteration of cell cycle distribution (Figure 4D). These findings suggested that the miR-181a-2-3p/STING axis was involved in the development and progression of colon cancer.

## DISCUSSION

Colon cancer is a serious public health issue, and its incidence and mortality rates have been increasing in recent years. Although cancer screening methods are rapidly evolving, the risk of developing colon cancer is increasing due to genetic risk factors, and environmental, dietary, and occupational exposures. Many patients are diagnosed with colon cancer during advanced screening, and survival largely depends on the stage at period of diagnosis [15]. Colon cancer has a high recurrence rate after surgical treatment, as evidenced by local recurrence, distant metastases, or heterochronic colorectal lesions. Other treatments, such as chemotherapy and radiotherapy, are associated with risks, such as complications have associated risks of complications.

Studies have shown that miRNA or miRNA expression profiles may be useful biomarkers for the diagnosis of colon cancer [16]. For example, miR-31 is highly expressed in the early stage of colon cancer, and transfection with miRNA-31 inhibitor was found to have anti-tumor effects with diagnostic and therapeutic value for colon cancer [17]. Therefore, miRNA has certain diagnostic and therapeutic value. Twenty-one miRNA profiles in the serum of stage IV colon cancer patients could distinguish between stage I or II [18]. Nine miRNAs were overexpressed in the plasma of colon cancer patients and were able to distinguish between colon cancer patients and controls [19]. In our study, we found that miR-181a-2-3p was lowly expressed in both colon cancer tissues and cell lines; thus, suggesting a potential role for miR-181a-2-3p in colon cancer.

In addition, miRNA therapy may be a powerful tool for colon cancer prevention and treatment. miRNA therapeutic strategies include inhibition of oncogenic miRNAs and restoration of tumor suppressor miRNAs. Several studies have shown that altered expression of oncogenic miRNAs is a causative factor in colon carcinogenesis. For example, overexpression of miR-494 inhibits the expression of APC, an inhibitor of the β-catenin signaling pathway, promoting cell proliferation and tumorigenesis in colon cancer [20]. Upregulation of miR-937 was significantly associated with lymph node metastasis and TNM staging. High expression of miR-937, lymph node metastasis and TNM stage were significantly correlated [21]. Serum miR-200c can be used as a predictive marker for lymph node metastasis in colon cancer and is more accurate than serum CEA levels or pathological staging, and it can be used to predict patient prognosis and early recurrence [22].

In addition, research has demonstrated that miRNAs have the potential to serve as therapeutic targets in colon cancer. In colon cancer, miR-212 functions as it exerts a tumor suppressor effect by directly targeting PIK3R3 and modulating regulating the AKT/mTOR signaling pathway [23]. In colon cancer tissues and cell lines, the expression of miR-138 is down-regulated in colon cancer tissues and cell lines, whereas overexpression of miR-138 suppresses colony formation, migration, and invasion of colon cancer cells *in vitro* [24]. In addition, these miRNAs play a regulatory role in radiation resistance in colon cancer. The expression of miR-375-3p was downregulated in colon cancer tissue cells, and the transplanted tumor experiment xenograft tests demonstrated that the therapeutic impact effect of combined miR-375+5-FU/NPs was much higher than that of therapy alone [25]. In colon cancer cell lines SW480 and HT29, overexpression of miR-181a-2-3p decreased cell proliferation and migration, enhanced cell apoptosis, and disrupted the cell cycle. Additionally, miR-181a-2-3p reduced the proliferation of colon cancer *in vivo*. In colon cancer, miR-181a-2-3p may act as a tumor suppressor.

Dual luciferase assay showed that miR-181a-2-3p had a targeting relationship with STING. STING (also known as TMEM173, ERIS, MITA, or MPYS) is located in the endoplasmic reticulum and initiates phosphorylation and activation of the transcription factor IRF3, which can enter the nucleus to promote transcription of inflammatory genes [26]. Our study found that increased STING expression partially counteracted the effects of miR-181a-2-3p overexpression on colon cancer cell lines, including inhibition of proliferation and migration, promotion of apoptosis, and altered cell cycle distribution. This suggests that the miR-181a-2-3p/STING axis is involved in the process of colon carcinogenesis and progression. STING enhances the antitumor effects of radiation and chemotherapy on cancer cells. Many antitumor chemotherapeutic agents and chemotherapy drugs induce cytotoxicity by damaging chromosomal DNA, which can be perceived by STING; thus, triggering type I interferon responses [27]. STING expression is usually suppressed or absent in most cancers, but its expression shows cancer type-specific differences. Individual studies have shown that activation of STING promotes cancer metastasis [28].

## CONCLUSION

To sum up, our study identified miR-181a-2-3p as an effector of colon cancer development. Moreover, we revealed that overexpression of miR-181a-2-3p promotes progression of colon cancer through STING. Importantly, our findings offered novel insights into the underlying molecular mechanisms that provide a potential molecular mechanism of miR-181a-2-3p which participates in colon cancer progression and lay the foundation for further miR-181a-2-3p-targeting therapeutical approaches for colon cancer. Further treatment of colon cancer can be done by targeting miR-181a-2-3p.

## MATERIALS AND METHODS

### Culture and passaging of colon cancer cell lines

#### Cell recovery

Firstly, the cell laboratory was routinely sterilized by UV light sterilization for 30 min and the ultra-clean table was ventilated for about 10 min. Then, fresh complete culture medium was placed in a constant temperature water bath and kept at 37°C. About 10 ml of fresh complete medium was taken and placed in a sterile cell culture flask. Freeze tubes (HCT116, HT29, SW480, SW620, and FHC) were removed from the liquid nitrogen storage tank and quickly placed into a 37°C thermostatic water bath while shaking them continuously, and then they were transferred to the sterile operation table after they were thawed rapidly. Subsequently, the cell suspension was transferred into the prepared fresh complete medium and incubated in a CO_2_ cell incubator at 37°C. After 24 h of cell apposition, the medium was changed and the culture was continued while cell growth and apposition status were observed. Cell recovery: UV sterilization for 30 mins, cryotubes (HCT116, HT29, SW280, SW620, FHC) were removed from the liquid nitrogen tank, thawed rapidly in a 37°C constant temperature water bath, and the cell suspension was transferred to the prepared fresh complete medium and incubated in a 37°C 5% CO_2_ cell incubator. After 24 hours of cell adherence, the culture medium was replaced and the cell growth and adherence were observed.

#### Cell passaging

Colorectal cancer cells were observed under a microscope and the passaging culture was started when the cell density was 80–90%. After wiping hands with 75% ethanol, the original culture medium was discarded, 1 ml of PBS was added to the pipette and shaken gently, PBS was discarded, and the number of times of washing the cells (about 1–2 times) was chosen according to the turbidity of the cell culture medium. Further, 0.5 ml of 0.25% trypsin was added to the culture dish (trypsin just spread over the cell surface) and the cells were digested at room temperature for about 3 min; meanwhile, digestion of cells was observed under the microscope. In order to prevent excessive digestion of colon cancer cells, which may lead to poor cell activity or even lysis and death, the cell digestion time was strictly controlled. After the digestion level was appropriate, the same volume of medium as trypsin was added and blown until all cells fell off from the bottom of the culture dish. Harvested suspension was transferred to a centrifuge tube, centrifuged at 1000 rpm for 5 min, and the supernatant was discarded. About 7 ml of fresh medium was added to the culture dish beforehand. After the cells were centrifuged, 1 ml of DMEM medium containing 10% FBS was added to resuspend the cells. An appropriate amount of cell resuspension was added to the culture dish, shaken well, observed under the microscope, and placed in the incubator. Cell passage: Passage began when the cells adhered to 80–90% of the wall. After disinfection for 30 minutes by ultraviolet irradiation, the original culture medium was discarded in the ultra-clean work table, and 5 ml PBS buffer was added to the Petri dish and then discarded. Trypsin was added and digested for 3 minutes. The harvested suspension was transferred to a centrifuge tube, centrifuged at 1000 rpm for 5 min, the supernatant was discarded, resuspended by adding fresh medium, dispensed into two or more Petri dishes, and cultured for 2–3 passages before subsequent experiments.

### Cell transfection

One day before transfection, SW480 and HT29 colon cancer cells were obtained in a positive growth state and logarithmic growth phase. The cells were inoculated in a 24-well plate and continuously cultured without the addition of antibiotics. (1) On the second day, transfection was started when the cell wall density reached about 50%. Then, RNA enzyme-free EP tubes were prepared, 50 pmol of miRNA mimics and 50 μl of serum-free and antibiotic-free Opti-MEM culture medium were added to the EP tubes and mixed gently (so that the final concentration of miRNA inhibitor was 100 nM), and they were allowed to stand for 5 min at room temperature. (2) Further, 1 μl of Lipofectamine 2000 and 50 μl of serum-free and antibiotic-free Opti-MEM culture solution were placed into a new EP tube, mixed gently, and allowed to stand at room temperature for 5 min. (1) and (2) were mixed gently (care was taken not to shake the mixture vigorously), and it was incubated for 20 min at room temperature. After 20 min, the liposome and miRNA mixture were added into the 24-well plate with a micromanipulator, and at least 3 wells were tested for each experimental group. Finally, DMEM culture medium was added to a final volume of 500 μl and incubated in the incubator for 6 h; the culture medium was discarded, fresh DMEM culture medium was added and the cells were continuously incubated for 24 h, and then subsequent experiments were performed. SW480 and HT29 colon cancer cells in the logarithmic growth phase were used for transfection, and a 24-well plate was used as an example. Transfection began when the cell adhesion density reached about 50%. Two sterilized centrifuge tubes were labeled, and 50 μl miRNA mimics and 50 μl Opti-MEM medium were gently mixed (the final concentration of miRNA inhibitor was 100 nM), and left at room temperature for 5 minutes. 1 μl Lipofectamine 2000 and 50 μl Opti-MEM culture medium were placed in another centrifuge tube, mixed gently, and left at room temperature for 5 min. The reagents in the two centrifuge tubes were gently mixed (taking care not to shake the mixture vigorously) and after 20 min incubation at room temperature the mixture was added to a 24-well plate and at least 3 wells per experimental group were tested. Finally, DMEM medium with a final volume of 500 μl was added and incubated in the incubator for 6 h. Fresh DMEM medium was replaced, and subsequent experiments were performed after continued incubation for 24 h.

### Real-time reverse transcription polymerase chain reaction (qRT-PCR) assay

#### Extraction of total RNA

Briefly, 1 ml of Trizol was added per six-well plate cells and mixed well by repeatedly blowing several times to perform sufficient lysis of the adherent cells. The lysate was transferred to an EP tube without RNA enzyme and centrifuged for 10 min (4°C and 12000 rpm), and the supernatant was obtained after centrifugation. Then, 200 μl of chloroform was added to the supernatant after centrifugation, shaken vigorously for 30 s to mix well, and then left for 10 min at room temperature. The mixture was centrifuged for 15 min (4°C and 12000 rpm), and the supernatant was obtained after centrifugation. Afterwards, 300 μl of isopropanol was added to the supernatant and placed at −20°C for 1 hour. Then, it was centrifuged at 4°C for 10 min (12,000 rpm/min) and the supernatant was discarded after centrifugation. Further, 1 ml of pre-cooled 75% ethanol was slowly added along the EP tube, the RNA was washed and then centrifuged again at 4°C for 10 min (7500 rpm/min). After centrifugation, the supernatant was discarded and the EP tubes were naturally inverted and dried, and finally the RNA was fully dissolved in RNase-free water. The specimens with OD260/OD280 values of 1.8-2.2 were considered as ideal specimens.

#### Reverse transcription to cDNA

The reverse transcription reaction was performed according to the instructions of the GEMA qPCR Quantitation Kit. The reaction system was prepared on ice to a final total volume of 20 μl, added to an eight-linked tube, and amplified in a PCR instrument. The conditions were 16°C for 30 min, 42°C for 30 min, and 85°C for 10 min; and the final product was placed in ice water immediately after the reaction.

#### qRT-PCR assay

The amplification conditions were as follows: 95°C for 3 min, 40 cycles; 95°C for 12 s, and 58°C for 35 s. The relative expression levels of target miRNAs or genes were calculated using the 2^−ΔΔCt^ method with U6 or GAPDH as the internal reference.

After collection of cells, RNA was extracted using a total RNA extraction kit and reverse transcribed into complementary DNA (cDNA) using a reverse transcription kit. The fluorescence quantitative system of SYBR Green qPCR Master Mix was configured as follows: 2× SYBR Green Master Mix (10 μl), ROX (0.4 μl), primer (0.4 μl), cDNA (2 μl), and DEPC-H2O (6.8 μl). Amplification conditions were as follows: 40 cycles at 95°C for 3 min; the cells were incubated at 95°C for 12 s and 58°C for 35 s. The relative expression of target miRNA or gene was calculated using the 2^−ΔΔCt^ method using U6 or GAPDH as an internal reference.

Has-miR-181a-2-3p: RT primer: GTCGTATCCAGT GCAGGGTCCGAGGTATTCGCACTGGATACGAC GGTACA; Quantitative F primer: GCGACCACTGAC CGTTGAC; Quantitative R primer: AGTGCAGGG TCCGAGGTATT; The housekeeping gene U6: F primer: GCTCGCTTCGGCAGCACA; R primer: GAACGCTTCACGAATTTGCGTG.

### Transwell assay for cell migration

Firstly, cells were inoculated. Colon cancer cells in the logarithmic growth phase were digested by 0.25% trypsin (containing EDTA), inoculated in 12-well cell culture plates at a density of 5 × 10^5^/well, and cultured for 24 h. Cell transfection was performed, and 3 replicate wells were set for each group of treatment. The medium was discarded, the cells were washed twice using PBS solution and replaced with serum-free medium, and the cells were starved for 24 h. Serum-free medium was discarded, cells were washed twice with PBS solution, digested with 0.25% trypsin (containing EDTA), and digestion was terminated by adding a complete medium. Then, 12,000 rpm centrifugation was performed for 30 s. Subsequently, the supernatant containing trypsin was discarded. The cell precipitate was resuspended by adding serum-free medium and cells were counted using a hemocytometer plate; the cell density of each treatment group was adjusted to the same level (10,000–20,000 cells/well). The Transwell plates were taken out, and 10% FBS concentration medium was added to the lower chamber of the culture plate 600 uL/well, and 100 uL/well of cell suspension that had been adjusted to the same density was added to the upper chamber of the plate. Three replicate wells were set up at the same time for each treatment group and incubated for 24 h. A new 24-well cell culture plate was prepared by adding 600 uL of 4% paraformaldehyde to each well. The chambers were carefully removed from the previous step using forceps, carefully washed twice with PBS solution, and placed in 4% paraformaldehyde for 30 min. Subsequently, the chambers were carefully washed twice with PBS solution. Depending on the number of chambers used, 0.1% crystal violet staining solution (600 uL/well) was added to the remaining wells of the 24-well plate in advance. The lower chamber of the vial was dried face up, photographed in a bright field mode under an inverted microscope, and five representative fields of view per well were selected for counting.

Inoculated cells and cell transfection. Colon cancer cells in logarithmic growth phase were seeded in 12-well cell culture plates at a density of 5 × 10^5^ cells/well. After 24 hours of culture, cell transfection was performed. The cells were collected and resuspended in serum-free medium, and the cell density of each treatment group was adjusted to the same level (10,000–20,000 cells/well). The Transwell plate was removed and the culture plate was adjusted to the same density of cell suspension by adding 10% FBS concentration medium 600 uL/well to the lower chamber of the culture plate and 100 uL/well to the upper chamber of the plate. Three duplicate wells were set up simultaneously for each treatment group and incubated for 24 h. A new 24-well cell culture plate was prepared by adding 600 uL of 4% paraformaldehyde to each well. The chambers were carefully removed from the previous step with forceps, carefully washed twice with PBS solution, and placed in 4% paraformaldehyde for 30 min. Subsequently, the chambers were carefully cleaned twice with PBS solution. According to the number of chambers used, 0.1% crystal violet staining solution (600 uL/well) was added to the remaining wells of the 24-well plate in advance. The vials were dried face up in the lower chamber and photographed in bright-field mode under an inverted microscope, and five representative fields per well were selected for counting.

### Cell cycle detection by flow cytometry

When colon cancer cells grew to 60–70% confluence, transfection was performed. The cells were placed in the incubator for 6 h, removed, and replaced with complete medium, and then continuously cultured for 48 h. Cell fixation was performed as follows: 70% ethanol was chilled in advance, and it was added to the cells, gently mixed, and placed in a refrigerator at −20°C overnight. Centrifugation was performed at 1500 rpm for 2 min, the supernatant was discarded, and cells were washed twice with pre-chilled PBS. The bottom of the centrifuge tube was gently bounced to avoid formation of clumps of cells. Then, 0.5 mL of C iodide staining solution was added to each group of cell samples, pipetted slowly to aspirate the cell precipitate, and placed in a warm bath for 30 min at 37°C protected from light. The flow cytometer was tuned to 488 nm to detect red fluorescence, as well as light scattering.

First, cells were fixed: 70% ethanol was pre-frozen, added to the cells, mixed gently, and placed in a −20°C refrigerator overnight. The cells were centrifuged at 1500 rpm for 2 min, the supernatant was discarded, and the cells were washed twice with precooled PBS. Then, 0.5 mL of C iodide staining solution was added to each group of cell samples, and the cell precipitate was slowly aspirated out and placed in a dark 37°C warm bath for 30 min. Finally, the flow cytometer was adjusted to 488 nm to detect red fluorescence and light scattering.

### Detection of apoptosis by flow cytometry

The cells were collected from each group after transfection, washed twice with PBS, suspended in 1× Binding Buffer, and adjusted to 1 × 10^6^ cells/ml. Then, 5 μl of Annexin V-FITC and 5 μl of PI were added to the cell suspension, the cells were protected from light for 15 min, 1× Binding Buffer was added, and apoptosis was detected by flow cytometry within 1 h.

### Dual luciferase assay

Plasmid used in this experiment: pmirGLO dual luciferase plasmid.

#### Cell lysis

The cell medium was aspirated, washed twice with PBS, and 20 μl of 1× Cell Lysis Buffer was added to each well. The cells were left to dissolve at room temperature for 5 min. The cell lysate was blown and aspirated into a 1.5 ml centrifuge tube, centrifuged at 12000 g for 2 min at room temperature, and the supernatant was used for subsequent detection.

#### Firefly luciferase reaction detection

100 μl of Luciferase Substrate equilibrated to room temperature was added to the microplate, and 20 μl of cell lysis supernatant was carefully sucked into the microplate wells. After rapid mixing, the Firefly luciferase reporter gene activity was detected in a multifunctional microplate reader immediately.

#### Renilla luciferase reaction detection

100 μl of freshly prepared Renilla substrate working solution was added to the above reaction solution, and the reporter gene activity of Renilla luciferase was detected in the microplate reader immediately after rapid mixing. The sequence of pmirGLO was shown in Supplementary File 1.

### Bioinformatics analysis

After login to the TCGA database (http://ualcan.path.uab.edu/index.html), click on the TCGA analysis TCGA miRNA, after entering miR-181a-2-3p in the search box, select the tumor type as colon adenocarcinoma, and then click explore to appear the expression and survival map. The clinical information of colon cancer patients was shown in Supplementary Table 1.

### Statistical analysis

Statistical analysis was performed using GraphPad Prism 8. All experiments were repeated at least three times, and the results were expressed as mean + standard deviation (Mean ± SD). Two groups of continuous variables were compared using the independent samples *t*-test. Multiple groups of continuous variables were compared using one-way ANOVA analysis, and two-by-two comparisons were made using the Tukey’s method. A *P*-value < 0.05 indicated that the difference was statistically significant. GraphPad Prism 8 software was used for statistical analysis. All experiments were repeated at least three times, and the results were expressed as mean + standard deviation (mean ± SD). Independent sample *t*-test was used to compare continuous variables between the two groups. One-way ANOVA was used to compare multiple groups of continuous variables, and Tukey’s method was used for pairwise comparison. *P* < 0.05 was considered statistically significant. The processed data of miR-181a-2-3p from TCGA datasets were shown in Supplementary Table 2.

### Data availability

The data of this study are available from the corresponding author upon request.

## Supplementary Materials

Supplementary File 1

Supplementary Table 1

Supplementary Table 2
