# Supplementary material for: Hsa-miR-181a-2-3p inhibits the oncogenicity of colon cancer by directly targeting STING
Source: Aging (Albany NY). 2024 Aug 9;16(15):11729–43. doi: 10.18632/aging.206059 (PMC11346793; doi:10.18632/aging.206059)
Supplement: Supplementary File 1 [file aging-16-206059-s001.pdf]

SUPPLEMENTARY FILE

Supplementary File 1. The sequence of pmirGLO plasmid.

1 catgcaagct gatccggctg ctaacaaagc ccgaaaggaa gctgagttgg ctgctgccac
61 cgctgagcaa taactagcat aaccccttgg ggcggccgct tcgagcagac atgataagat
121 acattgatga gtttggacaa accacaacta gaatgcagtg aaaaaaatgc tttatttgtg
181 aaatttgtga tgctattgct ttatttgtaa ccattataag ctgcaataaa caagttaaca
241 acaacaattg cattcatttt atgtttcagg ttcaggggga gatgtgggag gttttttaaa
301 gcaagtaaaa cctctacaaa tgtggtaaaa tcgaatttta acaaaatatt aacgcttaca
361 atttcctgat gcggtatttt ctccttacgc atctgtgcgg tatttcacac cgcatacgcg
421 gatctgcgca gcaccatggc ctgaaataac ctctgaaaga ggaacttggt taggtacctt
481 ctgaggcgga aagaaccagc tgtggaatgt gtgtcagtta gggtgtggaa agtccccagg
541 ctccccagca ggcagaagta tgcaaagcat gcatctcaat tagtcagcaa ccaggtgtgg
601 aaagtcccca ggctccccag caggcagaag tatgcaaagc atgcatctca attagtcagc
661 aaccatagtc ccgcccctaa ctccgcccat cccgccccta actccgccca gttccgccca
721 ttctccgccc catggctgac taattttttt tatttatgca gaggccgagg ccgcctcggc
781 ctctgagcta ttccagaagt agtgaggagg cttttttgga ggcctaggct tttgcaaaaa
841 gcttgattct tctgacacaa cagtctcgaa ccaaaggctg gagccaccat ggcttccaag
901 gtgtacgacc ccgagcaacg caaacgcatg atcactgggc ctcagtggtg ggctcgctgc
961 aagcaaatga acgtgctgga ctccttcatc aactactatg attccgagaa gcacgccgag
1021 aacgccgtga tttttctgca tggtaacgct gcctccagct acctgtggag gcacgtcgtg
1081 cctcacatcg agcccgtggc tagatgcatc atccctgatc tgatcggaat gggtaagtcc
1141 ggcaagagcg ggaatggctc atatcgcctc ctggatcact acaagtacct caccgcttgg
1201 ttcgagctgc tgaaccttcc aaagaaaatc atctttgtgg gccacgactg gggggcttgt
1261 ctggcctttc actactccta cgagcaccaa gacaagatca aggccatcgt ccatgctgag
1321 agtgtcgtgg acgtgatcga gtcctgggac gagtggcctg acatcgagga ggatatcgcc
1381 ctgatcaaga gcgaagaggg cgagaaaatg gtgcttgaga ataacttctt cgtcgagacc
1441 atgctcccaa gcaagatcat gcggaaactg gagcctgagg agttcgctgc ctacctggag
1501 ccattcaagg agaagggcga ggttagacgg cctaccctct cctggcctcg cgagatccct
1561 ctcgttaagg gaggcaagcc cgacgtcgtc cagattgtcc gcaactacaa cgcctacctt
1621 cgggccagcg acgatctgcc taagatgttc atcgagtccg accctgggtt cttttccaac
1681 gctattgtcg agggagctaa gaagttccct aacaccgagt tcgtgaaggt gaagggcctc
1741 cacttcagcc aggaggacgc tccagatgaa atgggtaagt acatcaagag cttcgtggag
1801 cgcgtgctga agaacgagca gaccggtggt gggagcggag gtggcggatc aggtggcgga
1861 ggctccggag ggattgaaca agatggattg cacgcaggtt ctccggccgc ttgggtggag
1921 aggctattcg gctatgactg ggcacaacag acaatcggct gctctgatgc cgccgtgttc
1981 cggctgtcag cgcaggggcg cccggttctt tttgtcaaga ccgacctgtc cggtgccctg
2041 aatgaactgc aggacgaggc agcgcggcta tcgtggctgg ccacgacggg cgttccttgc
2101 gcagctgtgc tcgacgttgt cactgaagcg ggaagggact ggctgctatt gggcgaagtg
2161 ccggggcagg atctcctgtc atctcacctt gctcctgccg agaaagtatc catcatggct
2221 gatgcaatgc ggcggctgca tacgcttgat ccggctacct gcccattcga ccaccaagcg
2281 aaacatcgca tcgagcgagc acgtactcgg atggaagccg gtcttgtcga tcaggatgat
2341 ctggacgaag agcatcaggg gctcgcgcca gccgaactgt tcgccaggct caaggcgcgc
2401 atgcccgacg gcgaggatct cgtcgtgacc catggcgatg cctgcttgcc gaatatcatg
2461 gtggaaaatg gccgcttttc tggattcatc gactgtggcc ggctgggtgt ggcggaccgc
2521 tatcaggaca tagcgttggc tacccgtgat attgctgaag agcttggcgg cgaatgggct
2581 gaccgcttcc tcgtgcttta cggtatcgcc gctcccgatt cgcagcgcat cgccttctat
2641 cgccttcttg acgagttctt ctgagcggga ctctggggtt cgaaatgacc gaccaagcga
2701 cgcccaacct gccatcacga tggccgcaat aaaatatctt tattttcatt acatctgtgt
2761 gttggttttt tgtgtgaatc gatagcgata aggatcctct ttgcgcttgc gttttccctt
2821 gtccagatag cccagtagct gacattcatc cggggtcagc accgtttctg cggactggct
2881 ttctacgtaa tggtttctta gacgtcaggt ggcacttttc ggggaaatgt gcgcggaacc
2941 cctatttgtt tatttttcta aatacattca aatatgtatc cgctcatgag acaataaccc
3001 tgataaatgc ttcaataata ttgaaaaagg aagagtatga gtattcaaca tttccgtgtc
3061 gcccttattc ccttttttgc ggcattttgc cttcctgttt ttgctcaccc agaaacgctg
3121 gtgaaagtaa aagatgctga agatcagttg ggtgcacgag tgggttacat cgaactggat
3181 ctcaacagcg gtaagatcct tgagagtttt cgccccgaag aacgttttcc aatgatgagc
3241 acttttaaag ttctgctatg tggcgcggta ttatcccgta ttgacgccgg gcaagagcaa
3301 ctcggtcgcc gcatacacta ttctcagaat gacttggttg agtactcacc agtcacagaa
3361 aagcatctta cggatggcat gacagtaaga gaattatgca gtgctgccat aaccatgagt
3421 gataacactg cggccaactt acttctgaca actatcggag gaccgaagga gctaaccgct
3481 tttttgcaca acatggggga tcatgtaact cgccttgatc gttgggaacc ggagctgaat
3541 gaagccatac caaacgacga gcgtgacacc acgatgcctg tagcaatggc aacaacgttg
3601 cgcaaactat taactggcga actacttact ctagcttccc ggcaacaatt aatagactgg
3661 atggaggcgg ataaagttgc aggaccactt ctgcgctcgg cccttccggc tggctggttt
3721 attgctgata aatctggagc cggtgagcgt gggtctcgcg gtatcattgc agcactgggg

3781 ccagatggta agccctcccg tatcgtagtt atctacacga cggggagtca ggcaactatg
3841 gatgaacgaa atagacagat cgctgagata ggtgcctcac tgattaagca ttggtaattc
3901 gaaatgaccg accaagcgac gcccaaccgg tatcagctca ctcaaaggcg gtaatacggt
3961 tatccacaga atcaggggat aacgcaggaa agaacatgtg agcaaaaggc cagcaaaagg
4021 ccaggaaccg taaaaaggcc gcgttgctgg cgtttttcca taggctccgc ccccctgacg
4081 agcatcacaa aaatcgacgc tcaagtcaga ggtggcgaaa cccgacagga ctataaagat
4141 accaggcgtt tccccctgga agctccctcg tgcgctctcc tgttccgacc ctgccgctta
4201 ccggatacct gtccgccttt ctcccttcgg gaagcgtggc gctttctcat agctcacgct
4261 gtaggtatct cagttcggtg taggtcgttc gctccaagct gggctgtgtg cacgaacccc
4321 ccgttcagcc cgaccgctgc gccttatccg gtaactatcg tcttgagtcc aacccggtaa
4381 gacacgactt atcgccactg gcagcagcca ctggtaacag gattagcaga gcgaggtatg
4441 taggcggtgc tacagagttc ttgaagtggt ggcctaacta cggctacact agaaggacag
4501 tatttggtat ctgcgctctg ctgaagccag ttaccttcgg aaaaagagtt ggtagctctt
4561 gatccggcaa acaaaccacc gctggtagcg gtggtttttt tgtttgcaag cagcagatta
4621 cgcgcagaaa aaaaggattt caagaagatc ctttgatctt ttctacgggg tctgacgctc
4681 agtggaacga aaactcacgt taagggattt tggtcatgag attatcaaaa aggatcttca
4741 cctagatcct tttatagtcc ggaaatacag gaacgcacgc tggatggccc ttcgctggga
4801 tggtgaaacc atgaaaaatg gcagcttcag tggattaagt gggggtaatg tggcctgtac
4861 cctctggttg cataggtatt catacggtta aaatttatca ggcgcgattg cggcagtttt
4921 tcgggtggtt tgttgccatt tttacctgtc tgctgccgtg atcgcgctga acgcgtttta
4981 gcggtgcgta caattaaggg attatggtaa atccacttac tgtctgccct cgtagccatc
5041 gagataaacc gcagtactcc ggccacgatg cgtccggcgt agaggatcga gatctaccgg
5101 gtaggggagg cgcttttccc aaggcagtct ggagcatgcg ctttagcagc cccgctgggc
5161 acttggcgct acacaagtgg cctctggcct cgcacacatt ccacatccac cggtaggcgc
5221 caaccggctc cgttctttgg tggccccttc gcgccacctt ctactcctcc cctagtcagg
5281 aagttccccc ccgccccgca gctcgcgtcg tgcaggacgt gacaaatgga agtagcacgt
5341 ctcactagtc tcgtgcagat ggacagcacc gctgagcaat ggaagcgggt aggcctttgg
5401 ggcagcggcc aatagcagct ttgctccttc gctttctggg ctcagaggct gggaaggggt
5461 gggtccgggg gcgggctcag gggcgggctc aggggcgggg cgggcgcccg aaggtcctcc
5521 ggaggcccgg cattctgcac gcttcaaaag cgcacgtctg ccgcgctgtt ctcctcttcc
5581 tcatctccgg gcctttcgac ctgcagccca agcttggcaa tccggtactg ttggtaaagc
5641 caccatggaa gatgccaaaa acattaagaa gggcccagcg ccattctacc cactcgaaga
5701 cgggaccgcc ggcgagcagc tgcacaaagc catgaagcgc tacgccctgg tgcccggcac
5761 catcgccttt accgacgcac atatcgaggt ggacattacc tacgccgagt acttcgagat
5821 gagcgttcgg ctggcagaag ctatgaagcg ctatgggctg aatacaaacc atcggatcgt
5881 ggtgtgcagc gagaatagct tgcagttctt catgcccgtg ttgggtgccc tgttcatcgg
5941 tgtggctgtg gccccagcta acgacatcta caacgagcgc gagctgctga acagcatggg
6001 catcagccag cccaccgtcg tattcgtgag caagaaaggg ctgcaaaaga tcctcaacgt
6061 gcaaaagaag ctaccgatca tacaaaagat catcatcatg gatagcaaga ccgactacca
6121 gggcttccaa agcatgtaca ccttcgtgac ttcccatttg ccacccggct tcaacgagta
6181 cgacttcgtg cccgagagct tcgaccggga caaaaccatc gccctgatca tgaacagtag
6241 tggcagtacc ggattgccca agggcgtagc cctaccgcac cgcaccgctt gtgtccgatt
6301 cagtcatgcc cgcgaccccа tcttcggcaa ccagatcatc cccgacaccg ctatcctcag
6361 cgtggtgcca tttcaccacg gcttcggcat gttcaccacg ctgggctact tgatctgcgg
6421 ctttcgggtc gtgctcatgt accgcttcga ggaggagcta ttcttgcgca gcttgcaaga
6481 ctataagatt caatctgccc tgctggtgcc cacactattt agcttcttcg ctaagagcac
6541 tctcatcgac aagtacgacc taagcaactt gcacgagatc gccagcggcg gggcgccgct
6601 cagcaaggag gtaggtgagg ccgtggccaa acgcttccac ctaccaggca tccgccaggg
6661 ctacggcctg acagaaacaa ccagcgccat tctgatcacc cccgaagggg acgacaagcc
6721 tggcgcagta ggcaaggtgg tgcccttctt cgaggctaag gtggtggact tggacaccgg
6781 taagacactg ggtgtgaacc agcgcggcga gctgtgcgtc cgtggcccca tgatcatgag
6841 cggctacgtt aacaaccccg aggctacaaa cgctctcatc gacaaggacg gctggctgca
6901 cagcggcgac atcgcctact gggacgagga cgagcacttc ttcatcgtgg accggctgaa
6961 gagcctgatc aaatacaagg gctaccaggt agccccagcc gaactggaga gcatcctgct
7021 gcaacacccc aacatcttcg acgccggggt cgccggcctg cccgacgacg atgccggcga
7081 gctgcccgcc gcagtcgtcg tgctggaaca cggtaaaacc atgaccgaga aggagatcgt
7141 ggactatgtg gccagccagg ttacaaccgc caagaagctg cgcggtggtg ttgtgttcgt
7201 ggacgaggtg cctaaaggac tgaccggcaa gttggacgcc cgcaagatcc gcgagattct
7261 cattaaggcc aagaagggcg gcaagatcgc cgtgtaattc tagttgttta aacgagctcg
7321 ctagcctcga gtctagagtc gacctgcagg
